# Metagenome-assembled genome sequence of an uncultured *Roseburia* sp. generated from mouse fecal DNA from the International Space Station

**DOI:** 10.1128/mra.01047-25

**Published:** 2026-06-15

**Authors:** Adit Chaudhary, Xuanyi Lin, Martha H. Vitaterna, Benjamin Auch, Ivan Liachko, Stefan J. Green

**Affiliations:** 1 Genomics and Microbiome Core Facility, Rush University, Chicago, Illinois, USA; 2 Department of Neurobiology, Northwestern University, Evanston, Illinois, USA; 3 Phase Genomics Inc, Seattle, Washington, USA; 4 Division of Infectious Diseases, Department of Internal Medicine, Rush University 2461, Chicago, Illinois, USA; Nanchang University, Nanchang, Jiangxi, China

**Keywords:** spaceflight, gut microbiome, *Roseburia*, microgravity, long-read metagenomics

## Abstract

The effects of spaceflight stressors, such as microgravity, cosmic radiation, and confinement, on the host physiology and gut microbiome remain unclear. Here, we report the metagenome-assembled genome (MAG) sequence of an uncultured *Roseburia sp*. strain that showed a significant gravity dose response in the gut microbiome of mice during spaceflight.

## ANNOUNCEMENT

Mouse fecal pellets were obtained from the NASA Mouse Habitat Unit 8 (MHU-8) mission (https://osdr.nasa.gov/bio/repo/data/payloads/MHU-8) that aimed to characterize the influences of spaceflight-associated stressors on the gut microbiome and host physiology. Mice (C57BL/6J male, obtained from The Jackson Laboratory, ME, USA) on the ISS were housed under different artificially generated gravity levels ranging from full Earth’s gravity (1G) to 0.67G, 0.33G, and finally 0G or microgravity (1). Short-read shotgun assembly-based metagenomic analysis revealed that *Roseburia* sp. 1XD42-69 (NZ_RAZG01000000) was significantly negatively associated with gravity. *Roseburia* is a genus of gram-positive anaerobes within the family *Lachnospiraceae*, order *Eubacteriales*, class *Clostridia*, and phylum Bacillota.

To reconstruct this genome, we combined PacBio long-read HiFi sequencing (2) with ProxiMeta-based Hi-C sequencing (3). We selected two in-flight samples from a single mouse (0G condition) that showed the highest relative abundances for this bacterium (3.2% and 4.0% based on short-read metagenomics). DNA was extracted from fecal pellets using a DNA Stool 360 kit with a Chemagic 360 instrument (Revvity, Waltham, MA, USA). The manufacturer’s standard protocol was followed, except for the use of 1.067 mL of Lysis Buffer 1, 20 µL of protease, and beat beating of tubes twice for 30 s (with 2-min rest in-between) before incubation. For PacBio HiFi sequencing, genomic DNA (gDNA) was processed through the PacBio SRE XS kit for removal of fragments <5 kb in length (PacBio, CA, USA). Size-selected DNA was processed using seqWell’s LongPlex multiplexing kit (seqWell, MA, USA). LongPlex libraries were converted to PacBio SMRTBell 3.0 libraries, and sequencing was performed with SPARQ chemistry on a Revio instrument with a 30-h movie at the DNA Services Core at the University of Illinois at Urbana-Champaign. Circular consensus sequence generation was done using SMRTlink 25.1 with the following parameters: ccs --min-passes 3 --min-rq 0.99. The sequencing yielded 1,325,948 and 1,381,167 reads per sample, with an average read length of 6,223 bp.

Data from the PacBio libraries were pooled and utilized for *de novo* assembly using the Flye assembler (v2.9.6-b1802) with “--pacbio-hifi” and “--meta” options (4). The assembled contigs were utilized as the reference assembly for a Hi-C sequencing and genome binning approach (Phase Genomics, WA, USA). A Hi-C library was created using a ProxiMeta Hi-C Microbiome Kit v4.0 (5). Intact cells from two fecal samples were crosslinked using a formaldehyde solution, digested using the Sau3AI and MlucI restriction enzymes, and proximity ligated with biotinylated nucleotides to create chimeric molecules composed of fragments from different regions of genomes that were physically proximal *in vivo*. To recover DNA after the cross-linking step, a reverse crosslinking enzyme was added, and the reaction was incubated at 65°C for 1 h. After reverse crosslinking, the DNA was purified using Recovery Beads, and Hi-C junctions were bound to streptavidin beads. The streptavidin-bound Hi-C DNA was then washed to remove unbound DNA and prepared for paired-end deep sequencing using the ProxiMeta Library Preparation reagents. Sequencing was performed on an Illumina NovaSeq X plus system, generating 87,100,000 read pairs (2 × 150 bp). Long-read assemblies and Hi-C metagenomic sequence data were analyzed using the Phase Genomics bioinformatics portal.

Bioinformatics analyses were performed using default parameters, except where noted. Briefly, Hi-C reads were processed by fastp (v0.20.1) using default parameters for adapter trimming and read quality filtering. Trimmed Hi-C reads were then aligned to the PacBio assembly using BWA-MEM (v0.7.17) (6), with the −5SP options specified, and all other options default. SAMBLASTER (v0.1.24) (7) was used to exclude PCR duplicates. Alignments were filtered with samtools (v1.9) (8) using the -F 2304 filtering flag to remove non-primary and secondary alignments. Matlock was used to filter read pairs with MAPQ scores <20 (https://github.com/phasegenomics/matlock). Metagenome deconvolution was performed with ProxiMeta (3, 9), resulting in the creation of 26 high-quality bacterial genome bins or MAGs with >90% completion and <5% contamination/redundancy based on CheckM (v1.2.3) (10). This included a MAG classified as *Roseburia* sp. 1XD42-69 by the Genome Taxonomy Database Toolkit (GTDB-Tk v2.4.0) (11). The final MAG contained two contigs, with 98.5% completion and 4.7% redundancy/contamination based on CheckM v1.2.3 (Table 1).

**TABLE 1:** Genome characteristics for the *Roseburia* sp. metagenome-assembled genome in comparison with a representative genome

Genome characteristic	*Roseburia* sp. (this study)	*Roseburia* sp. 1XD42-69 (NZ_RAZG01000000)
Estimated size (Mb)	5.03	4.7
Estimated completion (%)	98.5	98.9
Estimated contamination (%)	4.7	5.3
Number of contigs	2	282
N50 (kb)	3250.4	65.2
GC percent	43.2	42.5
Predicted genes (Prokka)	5,227	5,018
Annotated genes (Prokka)	2,029	1,843

The MAG was further analyzed using the Microbial Genomes Atlas (MiGA) webserver (12). Briefly, the genome was represented by two large contigs (contig lengths: 3,250,405 and 1,783,394 bp), with a combined length of 5,033,799 bp and a 43.22% GC content. The genome has 99.6% average amino acid identity (AAI) with the representative strain (https://www.ncbi.nlm.nih.gov/datasets/genome/GCF_003612565.1/). Four copies of small subunit (16S) and large subunit (23S) ribosomal RNA genes were detected, consistent with the 4–6 rRNA operon copies reported for other *Roseburia* genomes (13). MiGA identified 55 tRNA elements, corresponding to all 20 canonical amino acids. Prokka was used for annotation of the assembled genome (14) and predicted 5,227 genes, with 2,029 of these genes annotated with a known function. The metrics for the *Roseburia* sp. genome assembled in this study are summarized in Table 1 and compared with the metrics for *Roseburia* sp. 1XD42-69. The assembled genome from our study provides a valuable resource for future work aimed at understanding the genetic and metabolic capabilities of this strain and potential influence on host health and physiology in spaceflight.

## Data Availability

This Whole Genome Shotgun project has been deposited at DDBJ/ENA/GenBank under accession no. JBSXMH000000000. The version described in this paper is version JBSXMH010000000. The raw reads associated with generating this genome are available on NCBI SRA under BioProject PRJNA1314805, and with accession nos. SRR35275447, SRR35275446, and SRR35275445.
